# Strengthening National Public Health Institutes for resilient health systems: evidence to inform policy and decision makers in the Eastern Mediterranean region

**DOI:** 10.3389/fpubh.2025.1745722

**Published:** 2026-01-12

**Authors:** Tareq L. Mukattash, Dalia Kashef Zayed, Ala'a B. Al-Tammemi

**Affiliations:** 1 Jordan Center for Disease Control, Amman, Jordan; 2 Department of Clinical Pharmacy, Faculty of Pharmacy, Jordan University of Science and Technology, Irbid, Jordan

**Keywords:** Eastern Mediterranean region, evidence-informed narrative synthesis, financing, governance, health resilience, policy action, public health institutes, workforce

## Abstract

National Public Health Institutes (NPHIs) are pivotal to health security, yet their performance could be constrained by complex, interlinked barriers. This paper examines these operational challenges, with a focus on the Eastern Mediterranean Region (EMR), and argues that strengthening NPHIs requires context-specific, cross-domain policy action rather than isolated technical fixes. Drawing on global and EMR-specific experiences, we undertook an evidence-informed narrative synthesis of selectively identified peer-reviewed literature, documented case studies, and regional reports, to map barriers and policy options across six interconnected domains: governance and organizational frameworks; financing and resource allocation; workforce and capacity building; data, surveillance, and infrastructure; and coordination, multisectoral collaboration, and communication. Each domain is further influenced by pervasive political, social, and cultural factors. Our synthesis indicates that in the EMR, where many countries face ongoing conflicts, economic instability, and governance fragmentation, these barriers collectively weaken national preparedness and response capacities. The paper highlights the need for targeted policy action to overcome these constraints, specifically, adopting robust legal frameworks that clarify and reinforce NPHIs' mandates, developing diversified and sustainable financing mechanisms, institutionalizing competency-based workforce frameworks, and embedding risk communication and community engagement into routine operations. Overall, strengthening NPHIs along these dimensions is not a technical luxury but a strategic investment in national security, economic stability, and regional health resilience. Our paper provides EMR governments and partners with a practical roadmap to move from reactive, donor-driven responses toward coherent, country-led public health systems capable of managing both current and future threats within increasingly complex risk and humanitarian landscapes globally.

## 1 Background

National Public Health Institutes (NPHIs) have emerged as pivotal components of global health security, tasked with surveillance, outbreak investigation, and implementation of evidence-based policies that protect population health (1). These institutions are expected to deliver public health functions, ranging from data generation through robust epidemiological surveillance to the coordination of emergency responses and the formulation of policy, yet their performance is frequently hindered by a range of systemic and operational barriers (2).

Beyond emergency response, NPHIs contribute significantly to strengthening long-term population health outcomes. They generate and synthesize data that informs policy, ensuring that decision-making is rooted in reliable evidence. This includes monitoring disease trends, conducting health assessments, and evaluating the impact of interventions, all of which support the design of effective public health policies and programs. Additionally, NPHIs foster multisectoral collaboration, particularly through the One Health approach, bridging human, animal, and environmental health sectors to address complex health challenges holistically (3).

NPHIs also serve as hubs for research, innovation, and workforce development. By building technical capacity, offering training, and establishing standardized protocols, they enhance the quality and resilience of national health systems (4–6). NPHIs' efforts to strengthen laboratory systems, harmonize surveillance protocols, and promote data sharing not only maintain national health security but also reduce health inequities and improve health outcomes across populations (4–7). Consequently, NPHIs reinforce the foundation of public health governance, ensuring that countries are not only prepared for emergencies but also capable of sustaining healthier, more resilient societies (4, 5, 8–10).

Globally, NPHIs differ widely in their stage of institutional maturity, degree of autonomy, and organizational structure, and these differences offer important reference points for many other NPHIs (2, 5, 6, 10, 11). Policy choices on NPHI autonomy, functional consolidation, subnational presence, and surveillance architecture should be tailored to national geography and population distribution, and to socioeconomic constraints that influence service reach, equity, and partner coordination; thus, strengthening NPHIs should therefore be guided by context-specific risk assessments and resilience objectives, not institutional mimicry (10–14).

**Figure 1:**
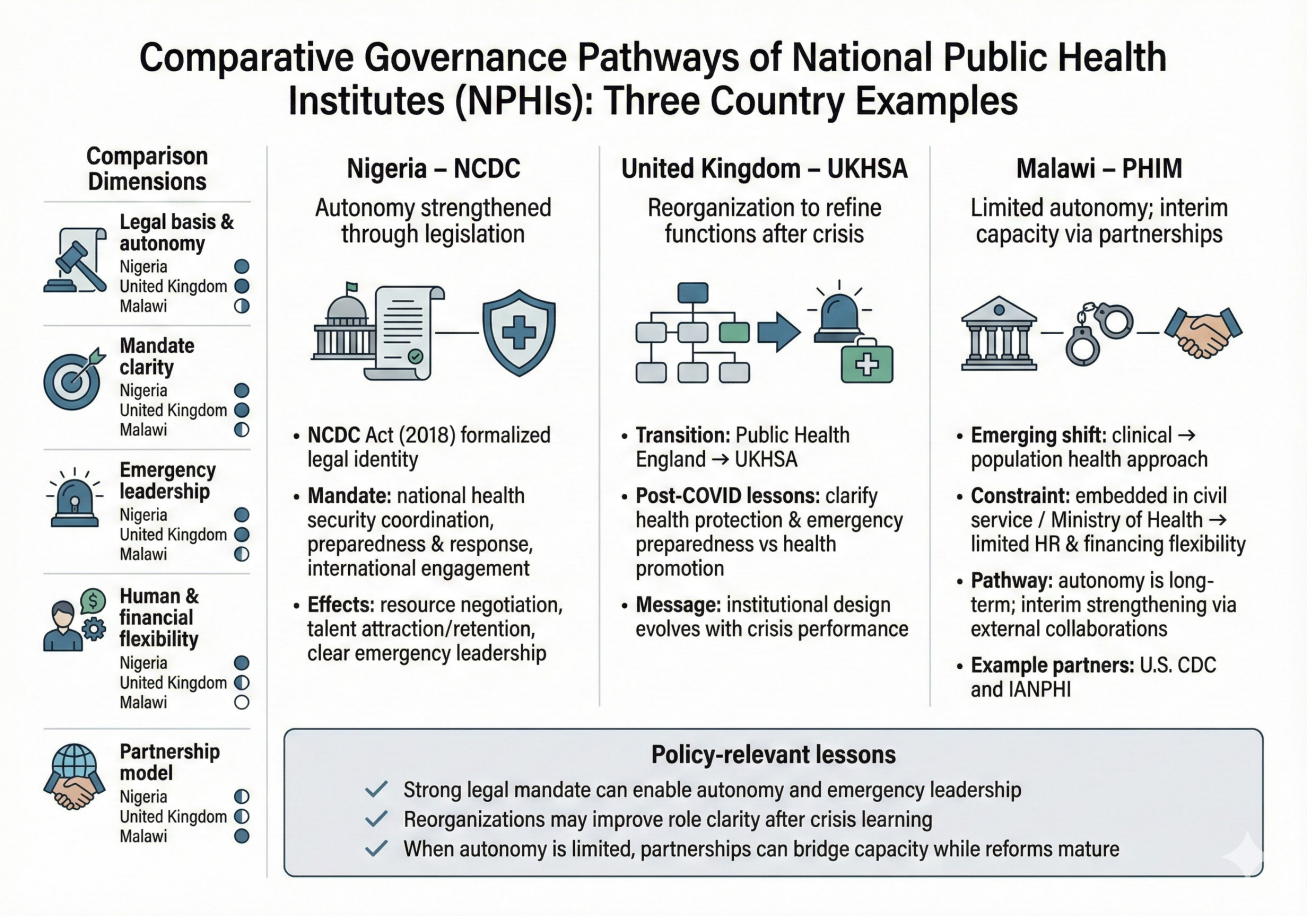
Three country examples of NPHI governance pathways and operational implications.

For instance, the Nigeria Centre for Disease Control (NCDC) is frequently cited as a success story in progressing to an autonomous parastatal institution, formally anchored by the NCDC Act (2018), which clarified its legal identity and mandate for national health security coordination, epidemic preparedness and response, and direct engagement with international partners (15). This legislative foundation has supported stronger resource mobilization, technical workforce retention, and clearer emergency leadership. In contrast, the United Kingdom (UK) transition from Public Health England to the UK Health Security Agency (UKHSA) highlights that even well-established systems face governance challenges; the post-COVID-19 reorganization sought to delineate health protection and emergency preparedness from wider health promotion functions, illustrating how institutional design evolves after crisis performance (16). In lower-income settings, such as in Malawi, the Public Health Institute of Malawi (PHIM) demonstrates the constraints of limited institutional independence when embedded within the civil service and Ministry of Health, particularly for human and financial flexibility; as legal autonomy can be a lengthy process, PHIM leveraged interim capacity building through external partnerships, including the United States Centers for Disease Control and Prevention (U.S CDC) and the International Association of National Public Health Institutes (IANPHI) to strengthen core functions (17). Further comparative governance pathways and operational implications are presented in Figure 1, noting that the selected cases are used to demonstrate different institutional pathways and design choices; they should not be interpreted as performance assessments or judgments about any individual NPHI.

These experiences validate that the barriers to effective NPHI functioning, particularly regarding political will and governance frameworks, are universal rather than region-specific. Collectively, this reveals that while the core functions of NPHIs including surveillance, emergency response, policy support, and workforce development are broadly similar across countries, the practical challenges of securing political commitment, adopting robust legal frameworks that clarify and reinforce NPHIs' mandates, and guaranteeing predictable, long-term financing are persistent and shared across both low- and high-income contexts.

In the Eastern Mediterranean Region (EMR), which comprises 22 member states and territories and has a population of nearly 745 million (18), political instability, recurrent humanitarian crises, and fragile health systems intensify operational challenges, while transparency around governance arrangements, mandates, and institutional roles remains compared to other regions (2).

While NPHIs are increasingly recognized globally, EMR-focused, policy-oriented syntheses that integrate global evidence with regional realities and translate these interlinked operational constraints into actionable reform options are highly needed. Accordingly, the aim of this perspective is to synthesize global evidence and EMR-specific experiences to identify the key challenges that may hinder the effective functioning and sustainability of NPHIs, both at the global level and within the EMR. Ultimately, this paper aims to strengthen health security and resilient health systems by informing nationally driven efforts to strengthen NPHIs and guide partner investments aligned with the 2030 Sustainable Development Goals (SDG), particularly SDG 3 (Good Health and Well-being), SDG 16 (effective, accountable institutions), and SDG 17 (Partnerships for the Goals).

## 2 Barriers to the effective performance and long-term sustainability of NPHIs

**Figure 2:**
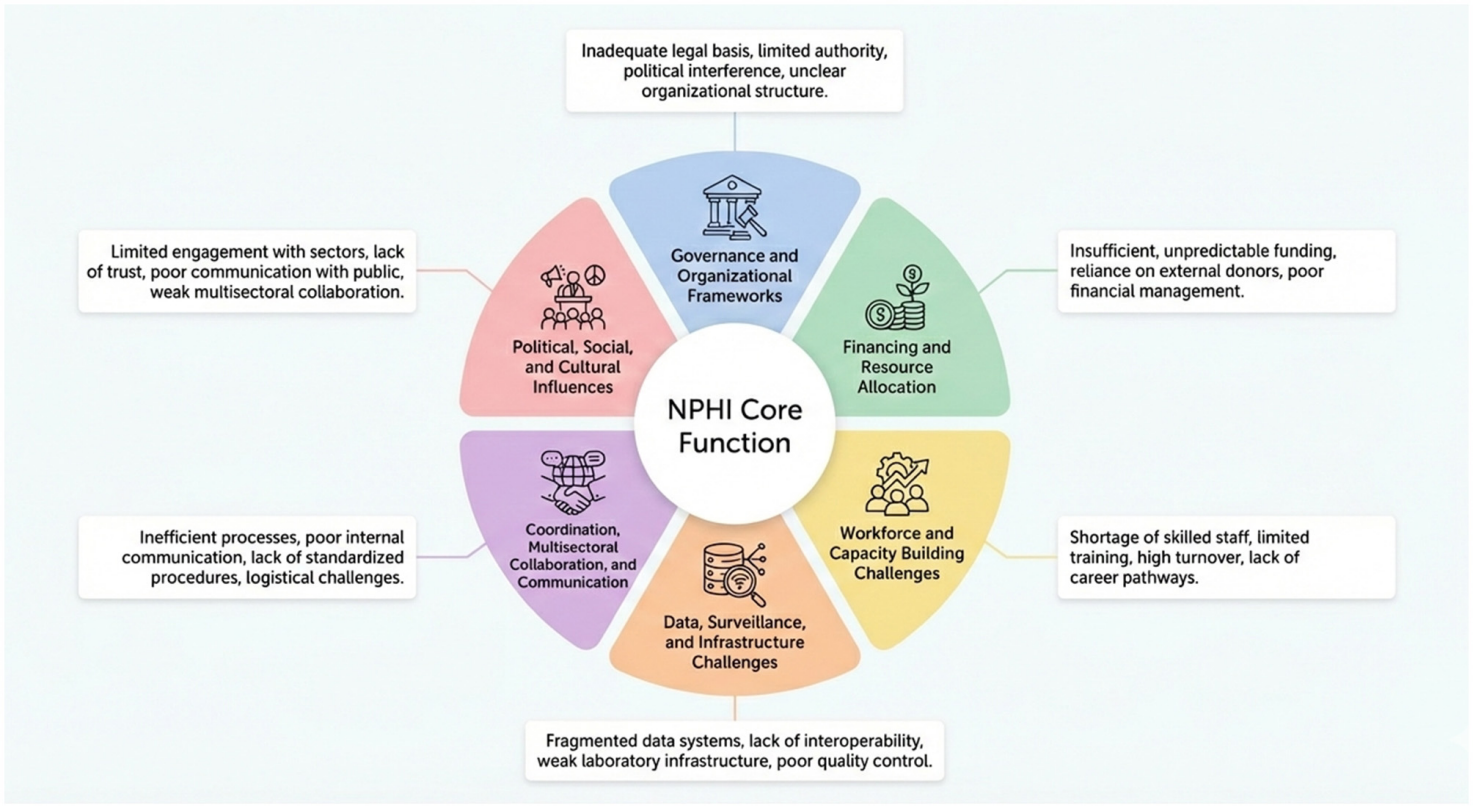
Barriers to the effective performance and long-term sustainability of NPHIs.

Given that fragmented governance, unstable financing, capacity shortages, suboptimal data systems and information sharing, and poor cross-sector coordination collectively weaken NPHIs and limit effective responses to emerging health crises (5, 8, 10, 11, 19, 20), Figure 2 summarizes the principal operational barriers that form the focus of this paper. Drawing on global evidence and EMR-specific experiences, we undertook an evidence-informed narrative synthesis of selectively identified peer-reviewed literature, documented case studies, and regional reports, to map barriers and policy options across six interconnected domains: governance and organizational frameworks; financing and resource allocation; workforce and capacity building; data, surveillance, and infrastructure; and coordination, multisectoral collaboration, and communication. Each domain is further influenced by pervasive political, social, and cultural factors.

### 2.1 Governance and organizational frameworks

The operability of NPHIs largely depends on their governance structures, which in many cases remain insufficiently adapted to address the evolving challenges of modern public health (21, 22). A recurrent challenge is the fragmentation of organizational authority, whereby institutes frequently function in advisory roles with restricted operational autonomy which is a scenario observed in several EMR countries but not unique to the region (23). This situation is further compounded by the overlapping mandates between NPHIs and ministries of health that result in unclear roles and diluted accountability (24). Moreover, NPHIs struggle with internal divisions and regional fragmentation exemplified by divided structures in conflict-affected countries, where differences in governance and political constraints inhibit coordinated responses (24, 25).

In addition, the absence of clearly defined legal frameworks and the limited enforcement authority afforded to NPHIs translate into challenges in the implementation and monitoring of public health legislation (25). Securing greater operational autonomy and instituting fit-for-purpose governance structures are imperative for overcoming these challenges. The governance challenges are further intensified by frequent leadership turnover, insufficient political commitment, and limited representation on key health policy committees, all of which significantly impact the influence of these institutes (25, 26).

### 2.2 Financing and resource allocation

Financial constraints represent one of the most pervasive barriers to the effective functioning of NPHIs globally (26). Many institutes remain caught in a cycle of reactivity, compelled to manage emergency responses in the absence of sustainable, long-term funding strategies (11, 27). Consequently, without sustainable financing frameworks, many institutes remain confined to reactive responses rather than proactive, sustained preparedness.

Across both high- and low-income contexts, inadequate government funding coupled with overreliance on external donors has fostered financial fragility, undermining strategic planning and the effective delivery of core public health functions (11, 19). Even where significant external financial support is available, reliance on short-term donor funding frequently leads to misalignment with national priorities and hinders the establishment of sustainable, integrated financing mechanisms (19).

In the EMR, where many countries are challenged by economic instability and political upheaval, the lack of sustainable domestic financing is a critical weakness that negatively impacts the resilience of public health systems (2, 28, 29). Without stronger government commitment and the establishment of robust, diversified funding models, the long-term sustainability of NPHIs remains in jeopardy (1). Furthermore, inconsistent financial support undermines critical functions such as laboratory operations and emergency preparedness, thereby heightening vulnerabilities during public health crises (1, 30). It is therefore essential to pursue financing reforms that strengthen domestic resource mobilization and lessen reliance on unpredictable external aid (25, 30).

### 2.3 Workforce and capacity building challenges

A critical factor underpinning the effective operation of NPHIs lies in the adequacy of human resources and the presence of a skilled, well-trained workforce (26, 27). The shortage of qualified personnel, including expert epidemiologists, laboratory experts, and public health administrators, severely constrains the capacity of these NPHIs to fulfill their mandates (31–36). High staff turnover, due in part to low remuneration, limited career development opportunities, and brain drain, further compromise institutional capacity (19).

This problem is most evident in low- and middle-income countries, where insufficient investment in capacity-building and the excessive workload carried by personnel across multiple roles place significant strain on the workforce (37, 38). In response, some institutions have initiated training programs, mentorship schemes, and collaborations with international entities to enhance workforce competencies; however, these initiatives remain limited in scope and sustainability (2, 38).

For workforce development to be effective, it must be supported by systematic investments in educational and training infrastructures that are tailored to local public health challenges (2). Moreover, the establishment of clear career pathways and professional certification programs is vital for retaining talent and cultivating a culture of continuous learning and innovation in the public health sector (23).

### 2.4 Data, surveillance, and infrastructure challenges

Robust surveillance and reliable data collection systems are fundamental for timely outbreak detection, informed policy development, and evidence-based decision-making in public health (23, 26). However, many NPHIs struggle with fragmented health information systems characterized by incomplete, inaccurate, and untimely data, which significantly restrict their operational effectiveness (27, 38). A persistent challenge is the absence of centralized, user-friendly, and interoperable data systems, which not only weaken routine disease surveillance but also delay the rapid deployment of resources during public health emergencies (1).

In addition, barriers to data sharing across public institutions and private health sectors constrain the ability to build a comprehensive epidemiological understanding (1, 2). Discrepancies in surveillance protocols, coupled with privacy and sensitivity concerns, contribute to a fragmented data landscape that limits timely public health responses (2). Tackling these challenges demands substantial investment in digital infrastructure, robust capacity building in data analytics, and the establishment of transparent, well-defined data governance frameworks. Equally critical is the need to overcome technical barriers and achieve full cross-sectoral integration of laboratory and field surveillance data, both of which are indispensable for reinforcing the resilience of public health systems (2).

### 2.5 Coordination, multisectoral collaboration, and communication

Effective public health response depends on robust internal coordination and strong collaborative networks between NPHIs, ministries, community, and external partners (21, 39). Many national institutes operate in silos, with limited mechanisms for interagency communication and coordination, thereby reducing their capacity to implement a unified public health strategy (22, 23). Fragmented collaborations between ministries, public and private sector stakeholders, and international organizations are frequently cited as impediments to coordinated outbreak responses (23, 24).

The establishment of formalized networks and joint coordination platforms is essential for strengthening multisectoral collaboration and ensuring the effective dissemination of knowledge and best practices across institutional boundaries (24). Moreover, in the context of pandemics, the lack of synchronized communication strategies and operational protocols can result in delayed responses and suboptimal allocation of resources (25). Furthermore, efforts must be made to ensure that collaboration is not merely top-down but actively involves field-level stakeholders to promote a bottom-up approach to problem solving.

### 2.6 Political, social, and cultural influences

The role of political and cultural factors in shaping the performance of NPHIs cannot be overstated, as these factors often dictate the level of institutional autonomy, resource allocation, and public trust (26). Political instability and frequent changes in government leadership compromise the continuity and effectiveness of public health initiatives, particularly in regions where public institutions are highly politicized (26, 27).

In the EMR, political unrest, conflicts, and corruption have been identified as significant barriers that undermine the capacity of NPHIs to function independently and assertively (11). Social and cultural barriers, including stigma around certain diseases and skepticism toward government-run institutions, further impede the effective dissemination of public health interventions (19). Cultural diversity and disparities in health literacy significantly influence the reception of public health messages and the effectiveness of surveillance activities (19).

In addition, mistrust between communities and government authorities can result in hesitation to share vital health information, creating additional obstacles for disease control and outbreak management (1, 37). Acknowledging and effectively addressing political and cultural factors is crucial for developing resilient, responsive public health systems that command widespread public trust and support (1).

The barriers outlined in this section are not theoretical; they are rooted in the complex socio-political realities of the EMR. Protracted armed conflicts in many EMR countries have fragmented governance, weakened health systems, and redirected domestic resources away from long-term system strengthening toward short-term humanitarian response (29). These dynamics have a major impact on the type, quality, and completeness of data that NPHIs in the region are able to generate and access.

At the social level, EMR countries are shaped by several common determinants, including rapid urbanization, a large youth population, increasing non-communicable disease burdens, significant internal displacement, and some of the highest refugee-to-host population ratios globally (40). Deep socioeconomic inequalities, uneven levels of health literacy, and rural–urban disparities, shape patterns of health risk, use of health services, and trust in public institutions, which is directly influencing how NPHIs can collect data, prioritize interventions, and communicate effectively with different population groups.

Also, in several settings in the EMR, communities depend heavily on parallel humanitarian and Non-Governmental Organization (NGO)-run service platforms, which often operate separate data systems that are not fully integrated into national surveillance. Consequently, this fragmentation limits NPHIs' ability to obtain a comprehensive, harmonized picture of population health (26).

Politically, many EMR countries have highly centralized decision-making, unstable or shifting political settlements, and frequent leadership turnover in both ministries of health and NPHIs (2). In fragile and conflict-affected settings, health is often closely linked to national security, with emergency response structures reporting directly to senior political or security authorities. While this can raise the profile of NPHIs and facilitate high-level engagement, it may also create sensitivities around data reporting, priority setting, and public communication, for instance, influencing how outbreaks with potential implications for tourism, trade, or politically sensitive areas are framed and disclosed (29). Collectively, these political and social factors help clarify why the evidence base on NPHI performance in the EMR is uneven and incomplete, and why it is largely determined by where data can be reliably and safely collected, an important limitation that policymakers must take into account in their decision-making and priority setting.

## 3 Discussion

This paper depicts a complex landscape where barriers to the effective functioning of NPHIs are multifaceted and intricately interconnected (2, 23). Governance and financing challenges are often at the core of these difficulties, but they are intricately linked with deficits in human resource capacity, data management, and multisectoral coordination (23). Strengthening NPHIs fundamentally requires alignment of their governance structures and legal mandates with core public health functions. Enabling legislation should explicitly confer authority for implementing surveillance, laboratory services, multisectoral coordination, risk assessment, public communication, and activation of incident management systems, while also ensuring clear leadership arrangements and formal representation within inter-ministerial health security mechanisms.

Governance arrangements need to reduce overlap with ministries of health, clarify accountability at national and subnational levels, and institutionalize learning cycles through after-action and intra-action reviews with time-bound follow-up. These measures are associated with faster response times, improved stewardship of essential functions, and greater uptake of evidence-informed policy. The persistent overreliance on external donor funding, coupled with political instability and fragmented legal frameworks, underscores a need for enhanced domestic commitment and the reform of public health financing models.

Against this backdrop, political and social conditions in the EMR do not simply form the context for NPHI work; they are central determinants of whether NPHI policies can be translated into operational reality. Comparative analyses of NPHIs in the region show that institutional structure, legal status, and political positioning strongly influence their ability to convene sectors, set technical standards, and secure resources for core public health functions (2).

In practical terms, one of the most important efforts is to consolidate the legal and governance foundations of NPHIs. Countries can strengthen implementation by enacting or updating dedicated NPHI legislation that clearly defines mandates, reporting lines, and accountability mechanisms; specifies the institute's role as the national hub for public health intelligence and risk assessment; and formalizes its seat on high-level inter-ministerial bodies responsible for health security and health system reform. Such laws can explicitly reference the International Health Regulations (IHR 2005) and essential public health functions, anchoring NPHIs within broader obligations for preparedness and response (41). Clarifying these political arrangements helps reduce mandate overlap with line departments, mitigates the risk of *ad hoc* restructuring, and gives NPHIs a more stable platform from which to coordinate implementation across the health system.

Also, political efforts must be accompanied by deliberate reforms in public financing and administrative rules that address problems arising from chronic under-investment and dependency on external funding. Regional health profiles document persistent under-investment in foundational public health capacities, with budgets constrained by competing clinical demands, emergencies, and debt-related pressures (42). To overcome this, governments can progressively establish protected domestic budget lines for core NPHI functions such as surveillance, laboratories, field epidemiology, and risk communication, and link these to medium-term expenditure frameworks rather than short annual cycles. This does not preclude external funding, but it reduces over-reliance on time-limited, donor-driven projects that fragment implementation.

In parallel, reforming public financial management and civil-service rules by simplifying procurement procedures for emergency and laboratory supplies, creating flexible mechanisms to retain critical technical staff, and setting minimum tenure periods for senior NPHI leadership can mitigate the operational disruptions associated with frequent leadership turnover and rigid hiring rules (29). These political and administrative choices directly affect whether NPHI policies remain aspirational documents or become fully resourced and executable programmes within the health system.

At the social level, entrenched inequities, displacement, and variable trust in public institutions shape how communities interact with health systems and how NPHI-led policies are implemented. Evidence shows that unequal living conditions, unemployment, and social exclusion drive large gradients in health status and access to services across and within EMR countries (43). To address the problems arising from these social determinants, efforts need to move beyond technical surveillance reforms to explicitly pro-equity implementation strategies. This includes designing NPHI policies that specify how surveillance, laboratory, and preparedness activities will cover refugees, internally displaced populations, informal settlements, and remote rural areas, and embedding requirements for disaggregated data (by geography, gender, socioeconomic status, and displacement status) into routine reporting templates. Formal agreements between NPHIs, ministries of health, and major humanitarian and NGO providers can help integrate parallel data systems into national surveillance platforms, ensuring that populations who primarily access non-state services are not invisible in national datasets (26).

Because political and social aspects are so decisive, trust-building and risk communication are essential levers for improving implementation. Studies on the political economy of health in the EMR conflict settings show how perceptions of politicization, corruption, or neglect can erode willingness to comply with public health measures and to share information with authorities (29). To counter this, NPHIs and ministries of health can institutionalize continuous social listening and community engagement processes, working with civil society, professional associations, and local leaders to co-design messages and feedback channels rather than relying only on top-down campaigns. This implies dedicated units or teams within NPHIs responsible for analysing social media trends, community feedback, and behavioral insights, and feeding these into policy adaptation and communication strategies, an approach increasingly recommended in EMR health emergency guidance (40). Over time, such practices can help rebuild social trust, reduce misinformation, and increase the acceptability of NPHI-led interventions across diverse social groups.

Additionally, a capable and stable workforce is critical for translating institutional mandates and financing into effective performance. National public health competency frameworks, aligned with core functions, should underpin workforce planning, define minimum staffing standards, and inform competency-based career pathways with clear progression criteria, appropriate remuneration, and protected time for continuing professional development (19, 39). Applied training programs in field epidemiology and laboratory sciences should be complemented by structured post-training placements at national and subnational levels, supervised practice, mentorship, and regular performance appraisal. Workforce retention is further enhanced when donor-funded positions are progressively integrated into the formal public sector establishment.

Furthermore, the limited operational autonomy of many institutes restricts their ability to leverage scientific evidence to inform policy, leading to reactive rather than proactive public health strategies (1, 38). Collaboration and communication gaps remain a significant challenge, as the lack of integrated systems and coordinated efforts not only delays response times during emergencies but also limits routine surveillance and prevention activities. In the context of the EMR, these challenges are exacerbated by conflict, fragile institutions, and cultural factors that impede both the collection of reliable data and effective community engagement (2).

To support both established NPHIs and those under development, integrating—and where already in place, sustaining and scaling—social listening, infodemic management, and community feedback mechanisms within routine operations can foster trust, improve uptake of public health measures, and expedite resolution of operational bottlenecks. Collectively, these actions elevate scientific independence, predictable financing, professionalized workforce development, interoperable data governance, and multisectoral coordination as central levers to improve prevention, detection, and response capacity, while simultaneously reinforcing day-to-day stewardship of essential public health functions in the EMR and beyond (25, 26, 44–48).

Addressing these barriers and challenges requires not only structural and operational reforms within individual institutes but also heightened regional and global collaboration to facilitate knowledge sharing, capacity-building, and the development of best practices that are context-specific (2). While global challenges are widely documented, the particular dynamics of the EMR require tailored strategies sensitive to its socio-political and economic contexts.

Fostering greater multisectoral engagement and ensuring that public health institutions have the autonomy and resources necessary to execute their mandates are critical to achieving long-term improvements in global health security. While considerable progress has been made in some settings, the persistent challenges identified in this paper reaffirm the critical importance of strengthening NPHIs by adopting contextually grounded policy actions and system-strengthening strategies aligned with established international standards.

NPHIs implementation efforts must acknowledge that EMR countries occupy very different positions along the spectra of fragility and resource availability, which creates distinct political and social constraints. High-income countries in the region, for example, can invest quickly in advanced laboratory networks and digital surveillance, while conflict-affected states face fragmented authority, damaged infrastructure, and strong humanitarian footprints (49). Rather than applying uniform solutions, NPHIs and their political sponsors can adopt a sequenced, context-specific approach, which means, in more fragile settings, priority efforts may include establishing a minimal but functional national surveillance core with integrated humanitarian data, negotiating pragmatic data-sharing arrangements with de facto authorities and the United Nations (UN) actors, and using Joint External Evaluation (JEE) and IHR as advocacy tools for incremental legal and financing reforms, while in more stable and better-resourced settings, efforts can focus on consolidating NPHI autonomy, institutionalizing integrated human–animal–environment health platforms, and using regional networks to mentor neighboring countries. Across this spectrum, the common thread is that political and social interventions (i.e. legislative reforms, financing decisions, equity-oriented design, and trust-building strategies) are not peripheral to NPHIs effectiveness; they are central to whether health system implementation aligns with NPHI mandates and delivers population-level impact.

To translate the findings into implementable priorities for the EMR, countries with established NPHIs as well as those in the process of establishing or reforming one should (i) formalize NPHI mandates and coordination arrangements through appropriate legal or administrative instruments that clarify roles across key sectors; (ii) establish an incident management interface with national emergency operations, and set clear information-sharing procedures during routine periods and humanitarian response; (iii) secure sustainable domestic financing by creating a protected budget line for core NPHI functions and adopting multi-year financing plans that include contingency mechanisms for surge response during shocks; (iv) implement competency-based workforce plans that ensure minimum staffing and surge capacity for epidemiology, laboratory services, and public health administration, supported by retention measures and deployable rosters capable of operating in remote and crisis-affected settings; and (v) operationalize interoperable surveillance–laboratory data exchange under defined data governance, enabling routine, timely reporting for priority threats and coordinated action across partners.

Finally, our paper urges governments, international agencies, and regional stakeholders in the EMR and beyond to prioritize public health as a core pillar of national development and security. Strengthening NPHIs along the aforementioned dimensions is not a technical luxury but a strategic investment in national security, economic stability, and regional health resilience. For EMR governments and partners, the findings provide a practical roadmap to move from reactive, donor-driven responses toward coherent, country-led public health systems capable of managing both current and future threats within increasingly complex risk and humanitarian landscapes globally.

## Data Availability

The original contributions presented in the study are included in the article/supplementary material, further inquiries can be directed to the corresponding author.
